# Diagnostic performance of radiomics model for preoperative risk categorization in thymic epithelial tumors: a systematic review and meta-analysis

**DOI:** 10.1186/s12880-023-01083-6

**Published:** 2023-08-29

**Authors:** Xue-Fang Lu, Tie-Yuan Zhu

**Affiliations:** 1 Dept. of Radiology, Renmin Hospital of Wuhan University, Wuhan, 430060 Hubei P.R. China; 2 Dept. of Thoracic Surgery, Renmin Hospital of Wuhan University, No. 238 Jiefang Road, Wuchang District, Wuhan, 430060 Hubei P.R. China

**Keywords:** Thymic epithelial tumor, Risk categorization, Radiomics, Meta-analysis

## Abstract

**Background:**

Incidental thymus region masses during thoracic examinations are not uncommon. The clinician’s decision-making for treatment largely depends on imaging findings. Due to the lack of specific indicators, it may be of great value to explore the role of radiomics in risk categorization of the thymic epithelial tumors (TETs).

**Methods:**

Four databases (PubMed, Web of Science, EMBASE and the Cochrane Library) were screened to identify eligible articles reporting radiomics models of diagnostic performance for risk categorization in TETs patients. The quality assessment of diagnostic accuracy studies 2 (QUADAS-2) and radiomics quality score (RQS) were used for methodological quality assessment. The pooled area under the receiver operating characteristic curve (AUC), sensitivity and specificity with their 95% confidence intervals were calculated.

**Results:**

A total of 2134 patients in 13 studies were included in this meta-analysis. The pooled AUC of 11 studies reporting high/low-risk histologic subtypes was 0.855 (95% CI, 0.817–0.893), while the pooled AUC of 4 studies differentiating stage classification was 0.826 (95% CI, 0.817–0.893). Meta-regression revealed no source of significant heterogeneity. Subgroup analysis demonstrated that the best diagnostic imaging was contrast enhanced computer tomography (CECT) with largest pooled AUC (0.873, 95% CI 0.832–0.914). Publication bias was found to be no significance by Deeks’ funnel plot.

**Conclusions:**

This present study shows promise for preoperative selection of high-risk TETs patients based on radiomics signatures with current available evidence. However, methodological quality in further studies still needs to be improved for feasibility confirmation and clinical application of radiomics-based models in predicting risk categorization of the thymic epithelial tumors.

## Introduction

Thymic epithelial tumors (TETs) are one of the most common primary tumors in the mediastinum, accounting for up to 50% of all anterior mediastinal neoplasms in adults [1, 2]. Incidental thymus tumors are almost always asymptomatic and appropriate serum markers are absent, so the clinician’s decision-making on treatment largely depends on imaging findings. Risk categorization of the thymic neoplasms is of great value in this situation.

According to the previous studies, the prognosis of thymic tumors largely depends on four prognostic factors: WHO histologic type, pathological stage, surgical margin status and pattern of treatment [3, 4]. In a large multiple-center cohort (n = 907), Liu et Colleagues from the ChART (Chinese Alliance for Research in Thymomas) confirmed that the WHO classification and T stage were independent prognostigators for recurrence of thymic tumors [5]. Patients with high-risk thymic tumors need more attention for timely surgery and comprehensive periopeartive treatment modality [6].

Jeong et al [7] tried to correlate the CT findings of thymic epithelial tumors with histologic classification as early as 2004, but found CT was of limited value in distinguish WHO histopathological subtypes. White and colleagues evaluated the efficacy of preoperative CT to predict the pathological stage of TETs. They concluded that the diagnostic accuracy rate of preoperative thoracic CT was two-thirds for TMN stage and less than 50% for Masaoka system [8]. One possible reason for the poor prediction performance is that subjective CT findings were evaluated in these studies, and disagreement was often seen when judging pericardial invasion, vascular invasion, lung invasion, pleural invasion and so on. Therefore, a more objective predictive model was urgently needed in this condition.

Radiomics refer to the use of computer technology to extract high-throughput quantitative features from medical images and transform the images into high-dimensional data, so as to reflect the biological characteristics in a non-invasive and objective way. Researchers have used radiomics models in TETs patients with regard to differential diagnosis, grading, staging or survival analysis [9]. As regard to differentiate risk subgroups of TETs, several studies demonstrated that radiomics model was a potential tool with acceptable diagnostic accuracy. However, there were limited relevant studies compared with other tumors, and the predictive power varied greatly, with AUC (area under curve) values of radiomics algorithms ranged from 70–90% [10, 11]. So we conducted a systematic review and meta-analysis to investigate the predictive performance of radiomics to act as an imaging biomarker for risk categorization of thymic tumors based on the published available literature. The results might serve as a benchmark for future prospective radiomics trials for clinical translation. This study followed the Cochrane Handbook for Systematic Reviews of Interventions and was conducted in accordance with the PRISMA (Preferred Reporting Items for Systematic Reviews and Meta-analysis) statement.

## Methods

### Search strategy

A systematic literature review of PubMed, Web of Science, EMBASE and the Cochrane Library was manually conducted from their establishment date until November 2022 to identify relevant reports. The databases were searched using the terms[(thymic or anterior mediastinal or thymoma or thymus neoplasm) and (radiomics or machine learning or deep learning or artificial intelligence or neural network)]. The search strategies incorporated the Medical Subject Headings terms and keywords. We omitted words, such as tumors, mass, lesions and so forth, in order to get more relevant articles to generate more power for analyzing this neglected issue. The search was limited to humans and performed with no language restrictions. References lists of the relevant articles were also screened.

### Study selection

The main outcome of our study was assessment of risk categorizations of thymic tumors. Risk categorizations include histologic subtypes classification and clinical/pathological stage classification. Histologic subtypes were classified into low risk (A,AB,B1) and high risk (B2,B3,C) groups according to WHO classification. While, stage classification was divided into early (I/II) and advanced (III/IV) stages according to TNM or Masaoka staging systems.

Criteria for inclusion in this study were as follow: (1) cohort or case-control studies; (2) patients with thymoma or thymic carcinoma proved by pathology; (3) all imaging-based (computer tomography, CT/magnetic resonance, MR/positron emission tomography-computer tomography, PET-CT) radiomics studies; (4) diagnostic outcomes (sensitivity, specificity, accuracy, etc.) were reported or could be calculated.

We excluded (1) non-original studies such as case reports, review, letters and commentaries; (2) studies involving other types of mediastinal tumors; (3) Studies with a replicated population.; (4) the diagnostic efficacy indicators were reported missing or could not be calculated.

### Data extraction and quality assessment

Two investigators (XF Lu and TY Zhu) independently screened the titles and abstracts of all relevant studies. The following data from each study was extracted: author, country, year of publication, study design, imaging modality, population for diagnostic accuracy, age, parameter extraction software and risk categorizations. And we made a quality evaluation to each study by quality assessment of diagnostic accuracy studies 2 (QUADAS-2) tools and radiomic quality score (RQS) [12, 13]. The QUADAS-2 tool includes four evaluation criteria: (a) patient selection; (b) index test; (c) reference standard; and (d) flow and timing. The RQS assessment included 16 aspects with 36 potential points. All disagreements between the two reviewers were resolved by discussion.

### Statistical analysis

According to QUADAS-2 standards, the RevMan 5.4 software was applied to fill in and draw the quality profiles included in this study. Meta-Disc 1.4 software was used to calculate threshold effects spearman correlation coefficient to evaluate the heterogeneity of threshold effects between studies. If there was no significant threshold effect heterogeneity between studies, Cochran’s Q test and I^2^ test were used to calculate the diagnostic odds ratio to evaluate the heterogeneity caused by non-threshold effects. I^2^ values were defined as no heterogeneity (0-25%), low heterogeneity (26-50%), moderate heterogeneity (51-75%) and high heterogeneity (76-100%) [14]. If P > 0.1 and I^2^ < 50%, the heterogeneity between studies was small, and fixed effects model was used for analysis. While if I^2^ > 50% and P < 0.1 indicating high heterogeneity, the random effects model was used for pooled analysis. The combined sensitivity, specificity, positive likelihood ratio, negative likelihood ratio, diagnostic odds ratio and 95% confidence interval (95%CI) were calculated. Summary receiver operating characteristic curve (SROC curve) was drawn and analyzed, and the area under the curve (AUC) was calculated. StataSE 16 software was used to draw Deeks’ funnel plot to determine whether there was publication bias between studies [15], and P > 0.05 was considered as no bias.

## Results

### Study selection

**Fig. 1:**
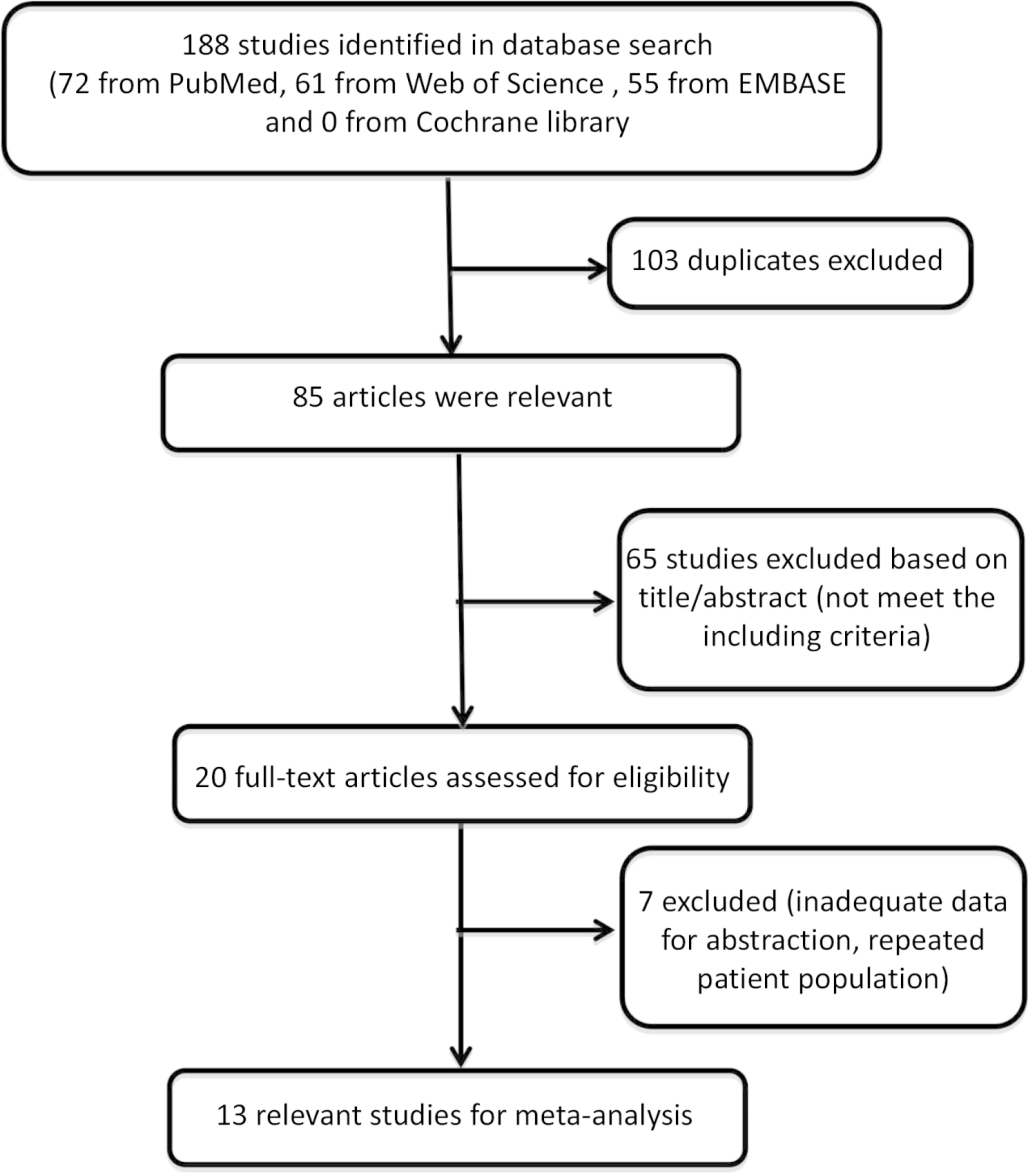
Flowchart of literature process

According to the retrieval strategy, a total of 188 relevant literatures were preliminarily obtained from the four databases, 103 duplicate literatures were eliminated, 65 were further eliminated by reading the title and abstract, 7 did not meet our inclusion criteria by two reviewer reading the full text, and 13 studies were finally included [16–28]. See Fig. 1 for the literature screening process.

### Study characteristics and quality assessment

A total of 2134 patients with thymic neoplasms were comprehensively analyzed in the included 13 literatures, including 1603 patients for histologic subtypes classifications (11 studies) and 531 patients for staging classifications (4 studies). The characteristics of retained studies were demonstrated in Table 1.

**Table 1:** Characteristics of included studies

Authors	Year of publication	Location	Study duration	Study design	Sample size	Age(y)	Image modality	Parameters extraction software	Total/included features	Risk classification
Sui (16)	2019	Changchun, China	2013.2–2018.3	Retrospective single-center	298	52.6	NECT/CECT	RadCloud	1029/12	A
Wang (17)	2019	Shenyang, China	2010.1–2018.10	Retrospective single-center	199	30–80	NECT/CECT	3D slicer v4.10	841/10	A,B
Chen (18)	2020	Guangdong,China	2009.2– 2019.3	Retrospective multiple-center	90	54 (19–81)	CECT	MATLAB 2016	10,394/8	A
Ren (19)	2020	Shanghai, China	2011.1–2019.4	Retrospective multiple-center	120	54.6 (24–77)	CECT	LIFEX v5.1.0	NA./43	A
Hu (20)	2020	Fuzhou, China	2009.1– 2018.12	Retrospective single-center	155	52.5 (23–79)	NECT + CECT	IBEX software	230/10	A
Xiao(21)	2020	Xi’an, China	2014.10–2018.11	Retrospective single-center	128	50.9 (22–75)	MRI	ITK-SNAP v3.6.0	4548/14	A
Blüthgen (22)	2021	Zurich, Switzerland	2000–2018	Retrospective single-center	62	57	CECT	PyRadiomics	1316/12	A,B
Dong (23)	2022	Nanchang, China	2017.7– 2022.3	Retrospective single-center	77	44.2	CECT	DARWIN Platform	558/13	A
Yu (24)	2022	Taiyuan, China	2012–2018	Retrospective single-center	130	54 (24–78)	CECT	RadCloud	1409/16	A
Araujo-Filho (25)	2022	New York, USA	2008.3–2019.7	Retrospective single-center	146	NA	CECT	METLAB	101/8	B
Tian (26)	2022	Tokyo, Japan	2001.1–2022.1	Retrospective single-center	124	61	NECT	3D slicer v4.10.2	851/15	B
Feng (27)	2022	Xi’an, China	2009.1–2018.5	Retrospective single-center	433	50	NECT	ITK-SNAP v3.6.0	1218/9	A
Nakajo (28)	2022	Kagoshira, Japan	20	Retrospective single-center	79	NA	PET	PyRadiomics v2.2.0	1131/3	A

CECT: contrast enhanced computer tomography; NECT: non-contrast enhanced computer tomography; MR: magnetic resonance; PET: positron emission tomography; NA: not available;

Risk classification: A, Low risk vs. High risk histological subtype thymic tumor; B, early stage vs. Advanced stage thymic tumor

**Fig. 2:**
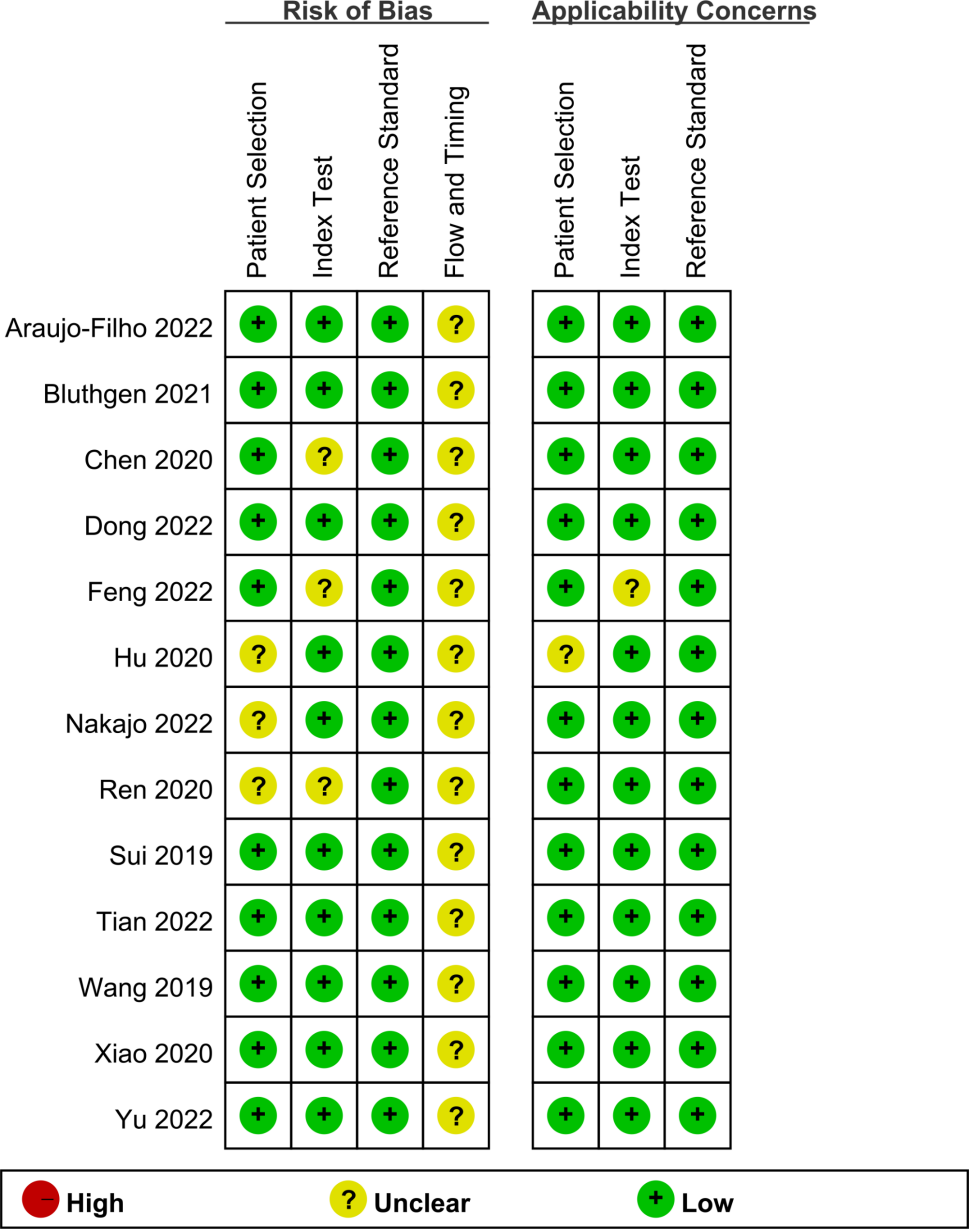
Methodological quality summary by QUADAS-2 for included studies

The data of the included 13 studies were complete. According to QUADAS-2 standard, the quality evaluation and mapping of all the included studies were conducted by RevMan 5.3 software. The main risk of bias came from the process and timing with unclear situation in all 13 studies. The specific quality evaluation results were shown in Fig. 2. For the RQS scale, mean score of included studies was 9.2 (range from 1 to 17). The mean RQS percentage was 25.6%(Table 2).

**Table 2:** The RQS scale for the included studies

Study	Image protocol	Multiple segmentations	Phantom study	Multiple time points	Feature reduction	Non-radiomics	Biological correlates	Cut-off	Discrimination	Calibration	Prospective	Validation	Gold standard	Clinical utility	Cost	Open Science	Total score (-8-36)	Percentage(%)
Sui	1	1	0	0	3	0	0	0	0	0	0	2	2	2	0	0	11	30.6
Wang	1	0	0	0	3	0	0	0	0	0	0	-5	2	0	0	0	1	2.8
Chen	1	1	0	0	3	1	0	1	1	1	0	4	2	2	0	0	17	47.2
Ren	1	1	0	0	3	1	0	0	0	0	0	3	2	2	0	0	13	36.1
Hu	1	1	0	0	3	0	0	0	0	0	0	-5	2	0	0	0	2	5.6
Xiao	1	0	0	0	3	1	0	0	1	1	0	2	2	2	0	0	13	36.1
Blüthgen	1	1	0	0	3	0	0	0	0	0	0	-5	2	0	0	0	2	5.6
Dong	1	1	0	0	3	1	0	0	0	1	0	2	2	2	0	0	13	36.1
Yu	1	0	0	0	3	0	0	0	0	1	0	2	2	2	0	0	11	30.6
Araujo-Filho	1	1	0	0	3		0	0	1	0	0	2	2	0	0	0	10	27.8
Tian	1	1	0	0	3	1	0	0	2	1	0	2	2	0	0	0	13	36.1
Feng	1	1	0	0	3	1	0	0	1	0	0	2	2	0	0	0	11	30.6
Nakajo	1	1	0	0	3	1	0	0	0	0	0	-5	2	0	0	0	3	8.3
Sui	1	1	0	0	3	0	0	0	0	0	0	2	2	2	0	0	11	30.6

### Diagnostic accuracy for histologic subtypes classification

**Fig. 3:**
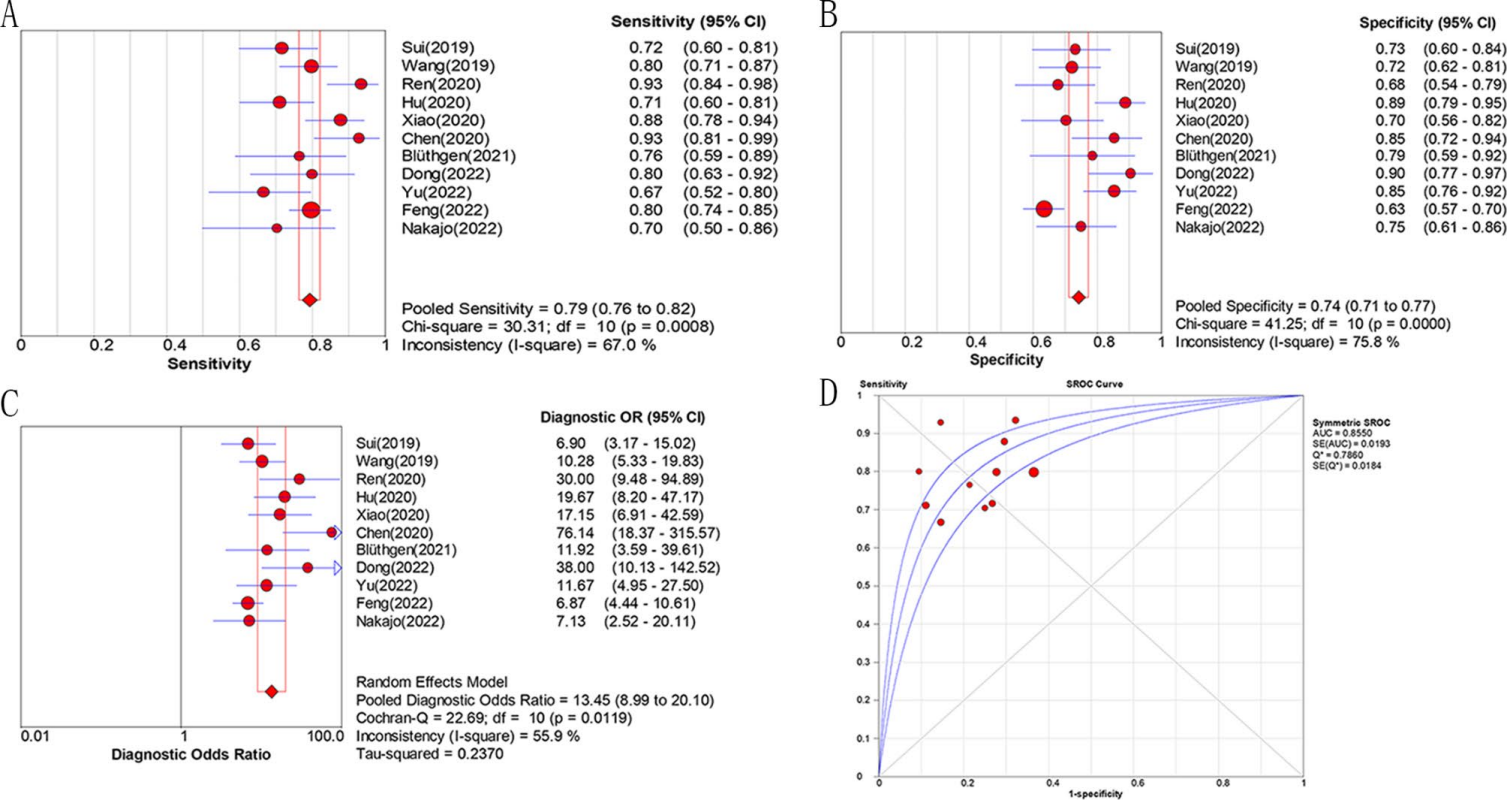
Diagnostic Accuracy for histologic subtypes classification: pooled sensitivity (**A**), specificity (**B**), diagnostic odds ratio (**C**) and AUC (**D**) of radiomics model

The heterogeneity test of the included 11 papers [16–24, 27, 28] showed that the spearman correlation coefficient of threshold effect was 0.273 (P = 0.417), indicating no heterogeneity caused by threshold effect. The heterogeneity of diagnostic odds ratio (DOR) was showed (Cochran’s Q = 22.69, P = 0.012, I^2^ = 55.9%) and the random effects model was used for meta-analysis. The results showed that the pooled sensitivity, specificity, positive likelihood ratio (PLR), negative likelihood ratio (NLR) and diagnostic odds ratio of radiomics model in the preoperative diagnosis of low/high risk thymic tumors were 0.794 (0.764–0.821), 0.743 (0.711–0.773), 3.392 (2.678–4.296), 0.285 (0.230–0.352) and 13.446 (8.995–20.099), respectively. The AUC value of summary receiver operating characteristic curve (SROC) is 0.855 (95% CI, 0.817–0.893), as shown in Fig. 3.

We performed sensitivity analyses to test how robust were the pooled results from the following aspects. 1)By calculating the pooled value using fixed effect model (Mantel-Haenszel), the value remain the same (AUC = 0.855); 2)By omitting one single study in each turn, and our results were stable consequently (AUC ranged from 0.843 to 0.864).

The discordance indexes (I^2^) of the above five pooled indicators were 67.0%, 75.8%, 65.4%, 49.4% and 55.9%, indicating mainly moderate heterogeneity of studies. Possible sources of heterogeneity were identified by meta-regression analysis model. The included indicators contained the number of patients included in the study (n < 100/≥100), country (Asia/Occident country), study design (single-center/multiple-center), imaging modality (NECT/CECT/MR/PET-CT), and machine learning. The source of significant heterogeneity was not identified by calculation of relative DOR with meta-regression (Table 3). As there was a tendency for significant differences in imaging modality (P = 0.06), the subgroup analysis was used to calculated the diagnostic accuracy of each modality (Table 4). The results showed that all subgroups had high diagnostic efficacy, but there were great differences among various modalities. The best diagnostic imaging was contrast enhanced CT with largest pooled AUC (0.873, 95% CI 0.832–0.914).

**Table 3:** Meta-regression of heterogeneity in included studies

Variables	r	P value	RDOR	95% CI
Number of patients included	0.164	0.792	1.18	0.23–5.96
Country	1.352	0.158	3.87	0.44–33.65
Study design	1.439	0.116	4.22	0.57–30.96
Imaging modality	−0.616	0.063	0.54	0.28–1.05
Machine learning	−0.114	0.843	0.89	0.20–3.99

RDOR: relative diagnostic odds ratio; CI: confidence interval

**Table 4:** Subgroup analysis of low/high risk thymic tumors by different preoperative imagings

Modality		No. of studies	Sensitivity	Specificity	AUC
CECT		8	0.784(0.745–0.820)	0.799(0.760–0.834)	0.873(0.832–0.914)
NECT		3	0.769(0.724–0.814)	0.681(0.599–0.762)	0.809(0.760–0.858)
MR		1	0.878	0.704	0.861(0.795–0.916)
PET		1	0.712	0.741	0.744(0.633–0.835)

No.: numbers; AUC: area under the receiver operating characteristic curve; CECT: contrast enhanced computer tomography, NECT: non-contrast enhanced computer tomography; MR: magnetic resonance; PET: positron emission tomography

### Diagnostic accuracy for stage classification

**Fig. 4:**
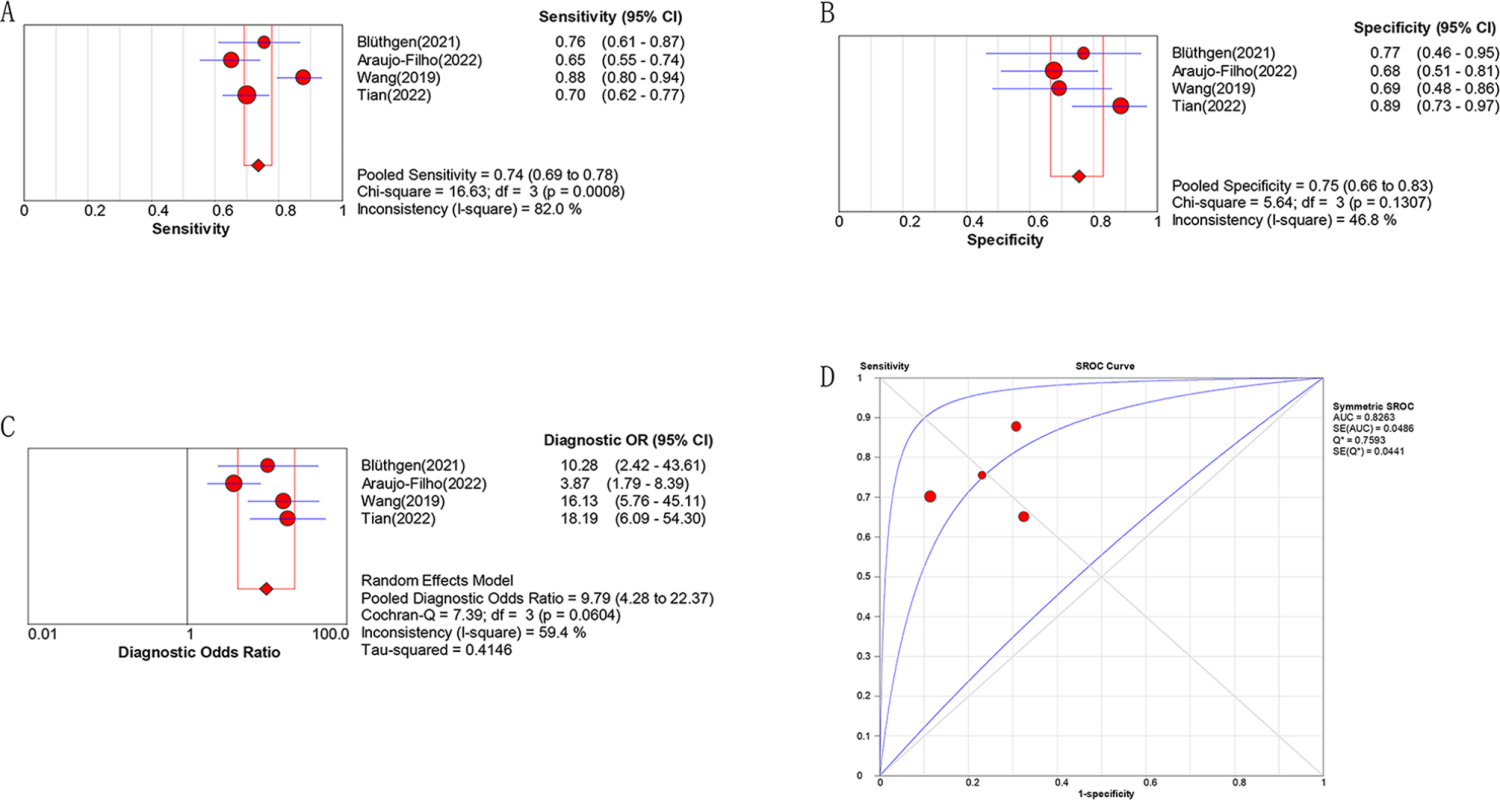
Diagnostic Accuracy for stage classification: pooled sensitivity (**A**), specificity (**B**), diagnostic odds ratio (**C**) and AUC (**D**) **o**f radiomics model

Four studies reported the diagnostic efficacy of radiomics for the staging of thymus tumors with NECT or CECT imaging [17, 22, 25, 26]. The spearman correlation coefficient assessment indicated no heterogeneity caused by threshold effect (P = 0.800). However the I^2^ statistic also showed moderate heterogeneity among the studies (I^2^ = 71.3%). Random effects model was used due to the heterogeneity test results of DOR. The combined sensitivity, specificity, PLR, NLR and DOR of radiomics model in the preoperative evaluation of early/advanced staging thymic tumors were 0.736 (0.691–0.778), 0.754 (0.665–0.830), 2.924 (1.845–4.635), 0.332 (0.226–0.487) and 9.791 (4.285–22.374), respectively. The AUC value of summary receiver operating characteristic curve was 0.826 (95% CI, 0.817–0.893), as shown in Fig. 4.

### Publication bias

**Fig. 5:**
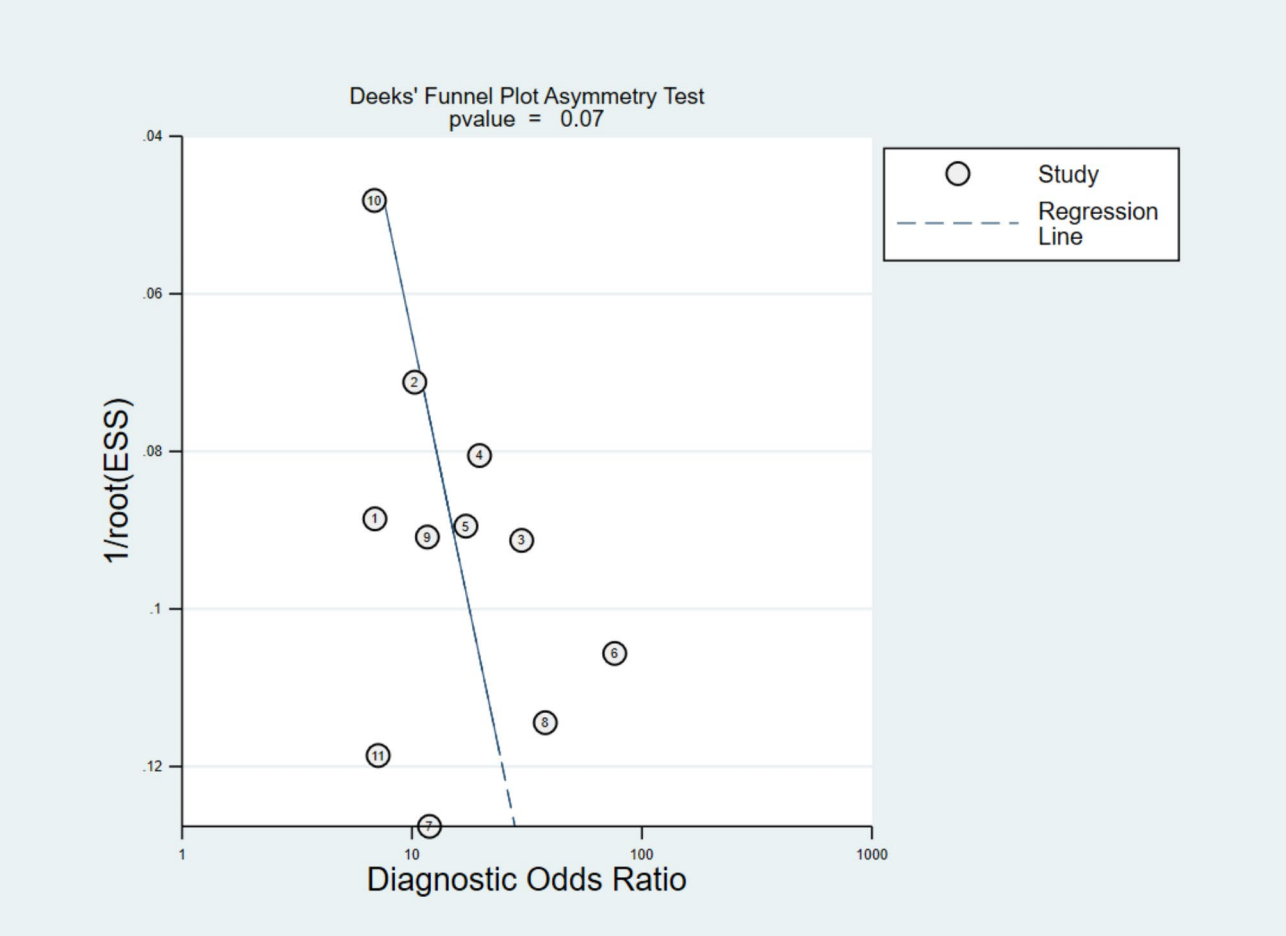
The Deeks’ funnel plot asymmetry test for subtypes classification

The Deeks’ funnel plot for subtypes classification was conducted and demonstrated in the Fig. 5. The figure did not show obvious asymmetry with P value > 0.05. This indicated that there was no significant publication bias in the included studies. Publication bias was not assessed for the staging classification, as the small number of studies included (n = 4) might lead to inconclusive funnel plot [29].

## Discussion

In the present systematic review, based on current evidence including more than two thousand patients, we found that radiomics has excellent diagnostic performance for risk categorization in thymic neoplasm patients. In addition, the highest predictive accuracy was based on contrast enhanced CT with a pooled AUC nearly 0.900 (0.873, 95% CI 0.832–0.914). Moreover, the researches of radiomics based on MR or PET-CT are still in its infancy, and need to be investigated and confirmed by further studies.

To the best of our knowledge, this is the first systematic review and meta-analysis to overview the diagnostic accuracy of preoperative radiomics model to predict risk classifications in TETs patients. We investigated the risk categorization from two aspects: high-risk histopathological subtypes and high-risk pathological staging, which both predicting complex treatments and worse outcomes [30]. We finally included a total of 13 predictive radiomics studies [16–28]. They were all published in the recent three years, with nearly half of them published in the last years (2022). Except for three studies from Switzerland, Japan and the United States [22, 25, 28], the other studies all came from different provinces of China [16–21, 23, 24, 26, 27]. This might be related to the low incidence of TETs and the difficulty of conducting multi-center radiomics studies. All the studies were retrospective nature, and only two [18, 19] included data from two medical centers. We used QUADAS-2 and RQS scale to evaluate the quality of literature. Probably because these studies were relatively new and had a basic similar design protocol, the quality of the literature was relatively high according to the QUADAS-2 tool. The main risk of bias came from flow and timing, as no studies have reported the time interval between radiomics and postoperative pathology results.There were also a small proportion of studies (3/13,23.1%) reported unclear patient selection [19, 20, 28] and index test [18, 19, 27], which might result in a small risk of deviation. A meta-regression was applied to investigate the radiomics-based prediction of low/high-risk WHO histologic subtypes, however, there was no statistical difference in the results. The sources of heterogeneity might be considered from the following aspects: (a) The scanning machines and scanning protocol varied in different institutions, which might influence the image acquisition; (b) Regions of interest were manually delineated in most researches, so there was a subjective component here; (c) Image feature extraction methods and extraction softwares were different; (d) There were multiple approaches for machine learning modeling, such as RF (random forest), SVM (support vector machine), kNN (k-nearest neighbor) and so on.We performed a subgroup analysis of the imaging modality, which showed a differential tendency (P = 0.06), but found that all the methods have good predictive performance, and chest enhanced CT remained the best model.

Because CT is the most commonly used examination of the chest, most of the radiomics were based on CT images. Wang et al. [17] Compared performance of radiomics signatures based on NECT and CECT for predicting high risk thymoma. Both radiomics showed excellent ability for risk categorization with high AUC (CECT 0.827 vs. NECT 0.801, P = 0.365). When compared with radiologists, only CECT-based radiomics signature showed statistically significant difference. However, other researchers (16) found that the radiomics features of the NECT scan outperformed CECT in risk grading for anterior mediastinal mass (AUC, CECT 0.741 vs. NECT 0.842). In our previous study, we found that the pooled AUC was slightly higher in the CECT radiomics signature than that of NECT (0.873 vs. 0.809), indicating that CECT-based radiomics might provide better diagnostic accuracy model. The underlying mechanism might be attribute to that the enhanced CT could better reflect the internal heterogeneity of TETs than the unenhanced computed tomography using texture analysis. Sui et al. [16] pointed out that some features, like tumor shape, shape-Spherical Disproportion, was selected from CECT, which probably because the enhancement scan highlighted the outline of the lesions.

In our present study, only one study [21] based on MRI radiomics and one based on PET-CT [28] were included. Researches on TETs based on these two types of radiomics were still in its infancy. Xiao et al. [31] Published a pilot study to explore the performance of MRI-based radiomics in risk stratification of TETs. The radiomics signatures demonstrated high AUC values of 0.880 and 0.948 for differentiating WHO high-risk subtypes and advanced staging. The outcomes of MRI-based radiomics studies were promising, particularly suitable for those who were allergic to iodine contrast agent or those who were afraid of radiation. Radiomics based on PET-CT for risk categorization in TETs patients was proved by few studies, however, the expensive cost limited its routine use.

Several non-radiomics indicators were included in the clinical combined radiomics models, such as, gender, age, myasthenia gravis and regular imaging findings (tumor size, pleural effusion, pericardial effusion, infiltration, etc.) [18, 19, 21, 23]. Although there was no statistical difference between the combined model and the radiomics model in the prediction efficiency, the absolute values were all improved in the studies. Further preoperative radiomics prediction studies were recommended to combine with clinical indicators.

Although this study provides the first comprehensive investigation of diagnostic performance of radiomics algorithm for risk categorization in TETs patients, there are also some limitations. Firstly, the included studies might be subject to some designed drawbacks, for instance, all studies were retrospective nature and the majority of studies had a small population. Secondly, the overall quality of the included studies was not optimal (mean RQS 25.6%), which might have potential influence of the subsequent analysis. Thirdly, heterogeneity was obvious among these included studies, though no source of heterogenenity was found by meta-regression. Last but not least, publication bias may be another major setback, because unreported non-significant radiomics models might be unavailable for analysis. However, the Deeks’ funnel plot suggested no significant evidence of publication bias in our study. According to above reasons, the clinical diagnostic TETs risk categorization tool based on radiomics should be rigorously conducted and evaluated in the future by prospective, multiple-center and well-design radiomics studies.

## Conclusions

In summary, this systematic review and meta-analysis shows promise for preoperative selection of high-risk TETs patients based on radiomics signatures with current available evidence. However, methodological quality in further studies still needs to be improved for feasibility confirmation and clinical application of radiomics-based models in predicting risk categorization of the thymic epithelial tumors.

## Data Availability

All data were collected or calculated from the published included studies.

## Abbreviations

AUC: Area under the receiver operating characteristic curve
CECT: Contrast enhanced computer tomography
MR: Magnetic resonance
NECT: Non-contrast enhanced computer tomography
NLR: Negative likelihood ratio
PET-CT: Positron emission tomography-computer tomography
PLR: Positive likelihood ratio
QRS: Radiomics quality score
QUADAS-2: Quality assessment of diagnostic accuracy studies 2
TET: Thymic epithelial tumor

## References

[1] Scorsetti M, Leo F, Trama A (2016). Thymoma and thymic carcinomas. Crit Rev Oncol Hematol.

[2] Tosi D, Damarco F, Franzi S (2022). Outcomes of extended surgical resections for locally advanced thymic malignancies: a narrative review. Gland Surg.

[3] Huang YY, Wu LL, Liu X (2021). Nomogram predict relapse-free survival of patients with thymic epithelial tumors after surgery. BMC Cancer.

[4] Alkaaki A, Al-Saud AA, Di Lena E (2022). Factors predicting recurrence in thymic epithelial neoplasms. Eur J Cardiothorac Surg.

[5] Liu H, Gu Z, Qiu B (2020). A recurrence predictive model for thymic tumors and its implication for Postoperative Management: a Chinese Alliance for Research in Thymomas Database Study. J Thorac Oncol.

[6] Kao TN, Yang PW, Lin MW (2020). Induction therapy followed by surgery for advanced thymic tumors. Asian J Surg.

[7] Jeong YJ, Lee KS, Kim J (2004). Does CT of thymic epithelial tumors enable us to differentiate histologic subtypes and predict prognosis?. AJR AM J Roentgenol.

[8] White DB, Hora MJ, Jenkins SM (2019). Efficacy of chest computed tomography prediction of the pathological TNM stage of thymic epithelial tumours. Eur J Cardiothorac Surg.

[9] Lee G, Bak SH, Lee HY (2018). CT Radiomics in thoracic oncology: technique and clinical applications. Nucl Med Mol Imaging.

[10] Ozawa Y, Hara M, Shibamoto Y (2019). The impact of radiomics in predicting oncologic behavior of thymic epithelial tumors. Mediastinum.

[11] Shen Q, Shan Y, Xu W (2021). Risk stratification of thymic epithelial tumors by using a nomogram combined with radiomic features and TNM staging. Eur Radiol.

[12] Whiting PF, Rutjes AW, Westwood ME (2011). QUADAS-2: a revised tool for the quality assessment of diagnostic accuracy studies. Ann Intern Med.

[13] Lambin P, Leijenaar RTH, Deist Tm (2017). Radiomics: the bridge between medical imaging and personalized medicine. Nat Rev Clin Oncol.

[14] Higgins JPT, Thompson SG, Deeks JJ, Altman DG (2003). Measuring inconsistency in meta-analyses. BMJ.

[15] Deeks JJ, Macaskill P, Irwig L (2005). The performance of tests of publication bias and other sample size effects in systematic reviews of diagnostic test accuracy was assessed. J Clin Epidemiol.

[16] Sui H, Liu L, Li X, Zuo P, Cui J, Mo Z (2019). CT-based radiomics features analysis for predicting the risk of anterior mediastinal lesions. J Thorac Dis.

[17] Wang X, Sun W, Liang H, Mao X, Lu Z. radiomics signatures of computed tomography imaging for predicting risk categorization and clinical stage of thymomas. Biomed Res Int 2019; 2019: 3616852. 10.1155/2019/3616852 PMC6558631 31275968

[18] Chen X, Feng B, Li C, Duan X, Chen Y, Li Z (2020). A radiomics model to predict the invasiveness of thymic epithelial tumors based on contrast–enhanced computed tomography. Oncol Rep.

[19] Ren C, Li M, Zhang Y, Zhang S (2020). Development and validation of a CT-texture analysis nomogram for preoperatively differentiating thymic epithelial tumor histologic subtypes. Cancer Imaging.

[20] Hu J, Zhao Y, Li M, Liu Y, Wang F, Weng Q (2020). Machine-learning-based computed tomography radiomic analysis for histologic subtype classification of thymic epithelial tumours. Eur J Radiol.

[21] Xiao G, Hu YC, Ren JL, Qin P, Han JC, Qu XY (2021). MR imaging of thymomas: a combined radiomics nomogram to predict histologic subtypes. Eur Radiol.

[22] Bluthgen C, Patella M, Euler A, Baessler B, Martini K, von Spiczak J (2021). Computed tomography radiomics for the prediction of thymic epithelial tumor histology, TNM stage and myasthenia gravis. PLoS ONE.

[23] Dong W, Xiong S, Lei P, Wang X, Liu H, Liu Y (2022). Application of a combined radiomics nomogram based on CE-CT in the preoperative prediction of thymomas risk categorization. Front Oncol.

[24] Yu C, Li T, Yang X, Zhang R, Xin L, Zhao Z (2022). Contrast-enhanced CT-based radiomics model for differentiating risk subgroups of thymic epithelial tumors. BMC Med Imaging.

[25] Araujo-Filho JAB, Mayoral M, Zheng J, Tan KS, Gibbs P, Shepherd AF (2022). CT Radiomic features for Predicting Resectability and TNM staging in thymic epithelial tumors. Ann Thorac Surg.

[26] Tian D, Yan HJ, Shiiya H, Sato M, Shinozaki-Ushiki A, Nakajima J. Machine learning-based radiomic computed tomography phenotyping of thymic epithelial tumors: Predicting pathological and survival outcomes. J Thorac Cardiovasc Surg 2022; S0022-5223(22)00797-8. 10.1016/j.jtcvs.2022.05.046 36038386

[27] Feng XL, Wang SZ, Chen HH, Huang YX, Xin YK, Zhang T (2022). Optimizing the radiomics-machine-learning model based on non-contrast enhanced CT for the simplified risk categorization of thymic epithelial tumors: a large cohort retrospective study. Lung Cancer.

[28] Nakajo M, Takeda A, Katsuki A, Jinguji M, Ohmura K, Tani A (2022). The efficacy of 18F-FDG-PET-based radiomic and deep-learning features using a machine-learning approach to predict the pathological risk subtypes of thymic epithelial tumors. Br J Radiol.

[29] Terrin N, Schmid CH, Lau J (2005). In an empirical evaluation of the funnel plot, researchers could not visually identify publication bias. J Clin Epidemiol.

[30] Turna A, Sarbay I (2020). Multimodality approach in treatment of thymic tumors. J Thorac Dis.

[31] Xiao G, Rong WC, Hu YC (2020). MRI Radiomics Analysis for Predicting the pathologic classification and TNM staging of thymic epithelial tumors: a pilot study. AJR Am J Roentgenol.

